# The Challenges and Lessons Learned Building a New UK Infrastructure for Finding and Accessing Population-Wide COVID-19 Data for Research and Public Health Analysis: The CO-CONNECT Project

**DOI:** 10.2196/50235

**Published:** 2024-11-20

**Authors:** Emily Jefferson, Gordon Milligan, Jenny Johnston, Shahzad Mumtaz, Christian Cole, Joseph Best, Thomas Charles Giles, Samuel Cox, Erum Masood, Scott Horban, Esmond Urwin, Jillian Beggs, Antony Chuter, Gerry Reilly, Andrew Morris, David Seymour, Susan Hopkins, Aziz Sheikh, Philip Quinlan

**Affiliations:** 1 Population Health and Genomics School of Medicine University of Dundee Dundee United Kingdom; 2 Health Data Research UK London United Kingdom; 3 Health Informatics Centre School of Medicine University of Dundee Dundee United Kingdom; 4 School of Medicine University of Nottingham Nottingham United Kingdom; 5 Patient and Public Representatives United Kingdom; 6 Public Health England London United Kingdom; 7 Centre for Population Health Sciences Usher Institute University of Edinburgh Edinburgh United Kingdom

**Keywords:** COVID-19, infrastructure, trusted research environments, safe havens, feasibility analysis, cohort discovery, federated analytics, federated discovery, lessons learned, population wide, data, public health, analysis, CO-CONNECT, challenges, data transformation

## Abstract

The COVID-19-Curated and Open Analysis and Research Platform (CO-CONNECT) project worked with 22 organizations across the United Kingdom to build a federated platform, enabling researchers to instantaneously and dynamically query federated datasets to find relevant data for their study. Finding relevant data takes time and effort, reducing the efficiency of research. Although data controllers could understand the value of such a system, there were significant challenges and delays in setting up the platform in response to COVID-19. This paper aims to present the challenges and lessons learned from the CO-CONNECT project to support other similar initiatives in the future. The project encountered many challenges, including the impacts of lockdowns on collaboration, understanding the new architecture, competing demands on people’s time during a pandemic, data governance approvals, different levels of technical capabilities, data transformation to a common data model, access to granular-level laboratory data, and how to engage public and patient representatives meaningfully on a highly technical project. To overcome these challenges, we developed a range of methods to support data partners such as explainer videos; regular, short, “touch base” videoconference calls; drop-in workshops; live demos; and a standardized technical onboarding documentation pack. A 4-stage data governance process emerged. The patient and public representatives were fully integrated team members. Persistence, patience, and understanding were key. We make 8 recommendations to change the landscape for future similar initiatives. The new architecture and processes developed are being built upon for non–COVID-19–related data, providing an infrastructural legacy.

## Introduction

In 2017, the UK Clinical Research Collaboration Tissue Directory and Coordination Centre (TDCC) [1] started a program to understand the processes required to allow federated discovery of biobanks based on the clinical and biomedical data they hold. There were many federated software solutions providing data discovery and data access, for example, TriNetx [2], i2b2 [3]/SHRINE [4], European Health Data & Evidence Network [5], iQVIA [6], and Leaf [7]. We chose the BC Platforms [8] solution for the TDCC work as they were a global leader in the field; had proven security and data confidentiality mechanisms built in; and most importantly, were willing to open their application programming interfaces such that we could implement new tools around their existing system. This program launched a beta version in February 2020 with 4 initial biobanks (Avon Longitudinal Study of Parents and Children [ALSPAC], National Institute for Health and Care Research [NIHR] Bioresource, Generation Scotland, and the Asymptomatic COVID19 in Education [ACE] cohort). The work also piloted the mechanisms in which to convert data to the Observational Medical Outcomes Partnership (OMOP) common data model (CDM) [9]. In March 2020, this was pivoted as part of the response to the COVID-19 pandemic, and the tools, know-how, and approaches were pivoted to the response to the pandemic.

The COVID-19-Curated and Open Analysis and Research Platform (CO-CONNECT) project was launched in November 2020, with funding from the Department of Health and Social Care UK Research and Innovation COVID-19 Rapid Response Initiative. At the time, new data were being generated by newly funded research projects and by the National Health Service (NHS) response to COVID-19, such as laboratory data from testing centers, in addition to routinely collected health data [10]. Research using these data was fundamental to the UK’s fight against the pandemic; however, it was challenging for researchers and public health agencies to find which data were being captured and by which organization or organizations [11]. This lack of visibility was further complicated by the 4 devolved nations within the United Kingdom each collecting data in different ways [12,13]. As the data were held by many different organizations, researchers had to contact multiple groups to determine if they held the data that could answer the key questions of policy interest. This took data analysis time away from researchers and time responding to the pandemic away from the organizations collecting the data. This was not a new issue [13], but its impact on timely research was made stark with COVID-19 [14-16]. Equally from a technical perspective, the challenges were identical to those sought to be solved by the earlier work of the TDCC.

Once researchers or public health agencies had found relevant data needed for their study, they required data sharing and data governance approvals. This took significant time, especially if data were required from multiple organizations, even considering the streamlined processes put in place during the pandemic (such as Control of Patient Information [COPI] notices) [17,18].

**Figure 1:**
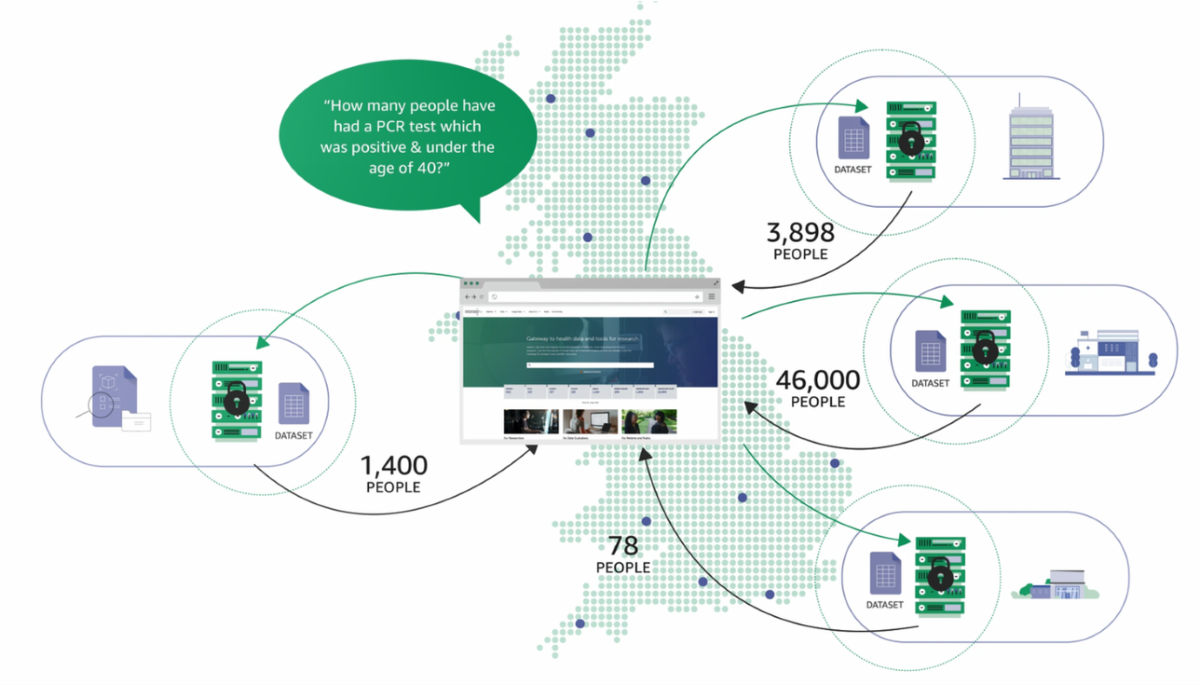
Schematic of federated querying from the Health Data Research Innovation Gateway. PCR: polymerase chain reaction.

CO-CONNECT was funded to respond to the needs of the research community to find and access data at pace while maintaining patient confidentiality [19]. The new CO-CONNECT infrastructure enables approved researchers and approved individuals from public health organizations to run aggregate-level, dynamic queries across UK-wide datasets in a federated manner. They can instantaneously discover the data that are available and run analyses with data remaining in situ (Figure 1). For example, “How many people have had a polymerase chain reaction (PCR) test that was positive and are younger than 40 years old?” An aggregate-level count of the people who meet the criteria is returned, for example, 102 people across 4 datasets. Queries are initiated from a single common website: the Health Data Research Innovation Gateway (Gateway) from within the Cohort Discovery Tool user interface [20]. Through a streamlined data governance application process and semiautomated pipeline, researchers can then request access to a linked, standardized, pseudonymized, project-specific subset of data to answer specific research questions. This “do once, reuse often” paradigm reduces the challenges and complexity of finding and making data available to answer urgent questions in the national interest.

The CO-CONNECT architecture (described in detail in Jefferson et al [21] and based on the early TDCC work) enables data sources to make their data discoverable and standardized while the data sources still maintain control over who is given access to the data. Each data source (termed CO-CONNECT data partners) hosts a secure virtual machine (VM) within their own network. The VM is separate from the location where identifiable data are stored. The data partners create a pseudonymized and standardized version of their data and deposit these within a database on the VM. Software installed within the VM runs queries received from the Gateway [22] against the database and returns an aggregate count of the number of individuals that meet the search criteria. The results from the queries run across all the data partners and are automatically collated and displayed within the Gateway for analysis by the user who initiated the query.

To configure the federated platform, we worked across 22 different data partners to on-board their data. Data partners included academic groups collecting data from a consented research cohort and national Trusted Research Environments (TREs) collecting population-wide, routinely collected data. We spent significant effort prior to grant submission to explain the benefits of the solution and included a representative from each data partner as a coapplicant on the proposal.

The Cohort Discovery tool is live within the Gateway, supporting authenticated researchers to find and access data—delivering the main output from CO-CONNECT [20]. However, there were significant challenges and delays along the journey. This paper describes the challenges faced and solutions developed and provides a series of recommendations to streamline future similar initiatives and access to UK-wide data for research and innovation.

## Challenges

### Addressing Cultural Norms During an Emergency Pandemic Response

**Figure 2:**
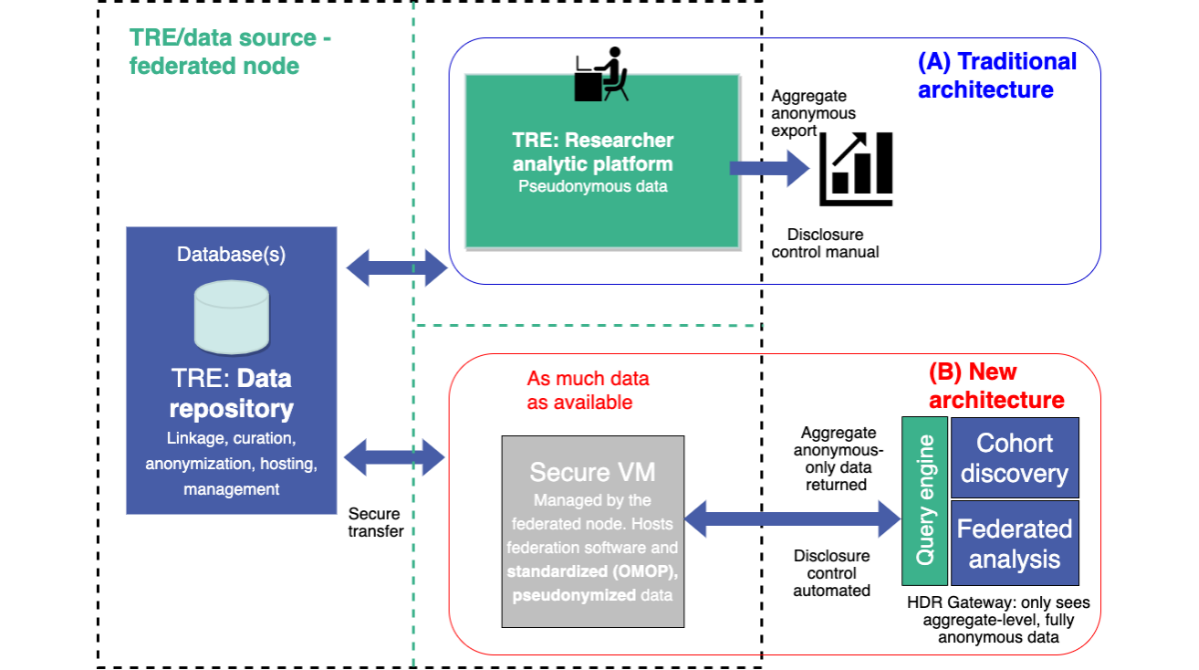
Comparison of traditional TRE architecture versus the new CO-CONNECT architecture. CO-CONNECT: COVID-19-Curated and Open Analysis and Research Platform; HDR: Health Data Research; OMOP: Observational Medical Outcomes Partnership; TRE: Trusted Research Environment; VM: virtual machine.

It was initially challenging for TREs to understand how the infrastructure we were building was different from the “normal” architecture of TREs. Figure 2A illustrates how TREs generally support research projects. TREs have an area within their infrastructure that hosts a data repository of population-wide information from a range of databases. For each research project, a project-specific set of data is copied from the data repository and placed in a separate area of their infrastructure where researchers can analyze the data. The project-specific set of data only includes the minimum amount of data that are needed to answer the specific scientific question and researchers can only access their specific dataset or datasets. There are stringent ingress and egress disclosure controls on the researcher area and there is no access to the internet. Only anonymous aggregate-level data can be exported from the researcher area, which are manually checked by trained TRE staff before release. Therefore, the primary role of the TRE is not to make data discoverable, nor to make it easy to link data across TREs, but to focus on providing a governance wrapper and keeping data secure within their given environments. Understanding the CO-CONNECT architecture required a mindset change for TREs, who are used to working in a set way.

For researchers, they are used to having collaborative projects, in which a group of researchers will come together, form a consortium, and generate and analyze data. Within a consortium, data may be shared between each of the entities involved, and data sharing agreements will be in place. The researchers will most likely have an agreed publication strategy, which also recognizes how involvement will equate to authorship and the research outputs. The reuse of data and the publishing of data are often only seen as something to do at the end of the project, as part of archiving or validating the results as a requirement from funders or journals. Therefore, a project that sought to make data discoverable before publication, even if just basic metadata, was a very different ethos and culture for academic researchers to operate.

Patient and public involvement and engagement (PPIE) have become a cornerstone of medical research to ensure the public voice is heard and indeed where public and patient members help to cocreate the solutions. Those with lived experience may be able to inform on the development of a new medicine or medical device or service, and therefore, PPIE on medically oriented projects are commonplace. CO-CONNECT was a very technical program in parts and we had a strong desire not to exclude our PPIE members from the technical design components so that we could ensure full transparency and scrutiny across all decisions. While much guidance on PPIE does exist, there was very little on how to embed PPIE into a software development team for example.

### Awareness of Data Discovery Approaches

Although there are many different solutions that provide data discovery and federated analysis, most of the data partners we worked with were unfamiliar with such solutions (Figure 2B). For both the academic and TRE teams, this meant quite a significant amount of effort in helping them to understand the core concepts: for example, for a researcher, how they could make data discoverable without sharing it and protecting their datasets before they had published. For TREs, this was about how the technical approach could be consistent with their existing security models and approaches. Federated and data discovery systems are not new, but it was clear that they were still not understood or known by a significant portion of the community. This was especially true when it came to local configuration and how these approaches could be demonstrated to be consistent with the five safes framework.

### Standardized Software to Profile Data Within Trusted Research Environments

CO-CONNECT uses the OMOP CDM [9] to enable federated queries with comparable answers across data partners. We needed metadata describing the datasets held by data partners to use as input to a tool that would convert their datasets into OMOP format [23-25]. Metadata are anonymous data about the data that are captured, for example, field names, ranges of data within the fields, and descriptions of what data are collected in each field. We sought to use the WhiteRabbit data profiling tool from Observational Health Data Science and Informatics to generate the metadata [26]. The academic community was largely happy to install this, but for the TREs that operate within a highly controlled environment, the software had to undergo several layers of security assessment to assure this open-source software to confirm that only metadata were shared (therefore, data sharing agreements are not required) and then to test the solution and confirm the outputs were not disclosive.

### Data Governance

#### Overview

Implementing the technological components of the project was relatively straightforward. However, although the data partners were supportive of the initiative, obtaining data governance approvals, especially for the national TREs, was a significant challenge. Most of the time spent with TREs was for them to understand the process and which groups needed to approve this new infrastructure and process. The actual governance approvals took a long time. In some cases, but not all, governance approval was a relatively short time in comparison to collaborating with the TREs to populate forms with the information they required prior to submission. There were several governance challenges.

#### No Existing Governance Framework

There was no process that we could simply apply to undertake the work; indeed, it was also not clear whether the CO-CONNECT team should be applying the process or simply helping each data partner to form an opinion. The design of the system meant that at no stage would any third party have access to the data. The questions asked within all data governance approval documents assumed that someone would be accessing the data. What we were doing was to provide support to enable each data partner to connect to a national data discovery framework, via software. Most TREs did not have a documented process to assess new software being installed within their environments for a research project (as it was not normally requested). They did have methods for assessing third-party provider software for their core systems, but then that process would normally have followed a procurement route, where a strategic decision and funding were made to redesign or develop parts of their infrastructure.

The academic-led studies were also supportive of the initiative but were not sure of the formal approaches to approve a project such as CO-CONNECT. The CO-CONNECT team did mandate that all data partners undertake a data protection impact assessment to ensure that each organization considers its involvement carefully. Overall, once data partners understood how the architecture worked, they could see the benefits of the solution and how security and patient confidentiality were protected, but the national TREs were unsure of how to say “yes.”

#### Bespoke Approaches Across Data Partners

Each data partner subsequently sought approval from the various committees to ensure all bases were covered. Each committee, for each data partner, had different document requirements, different formats, and different levels of detail. For some national TREs, 4 different forms were required to go to 4 different committees or approvers, with each form being >100 pages long. Drafting such information across many data partners took significant time.

#### Single Points of Failure for Approval of Data Governance Applications

Many data partners only had a single individual within their organization who could formally approve and sign off the data governance applications. The pandemic resulted in a much higher than usual number of applications, especially because of the temporary COPI notice in England [18], which resulted in an increase in the volume of research and data sharing requests. We were told in one instance that there was a 6-month wait for applications to be reviewed due to the backlog and only a single person with the authority to approve requests. The technical nature of the project meant that some institutions had preferred individuals who could review this type of application. This led to further bottlenecks when this individual was time-constrained or unavailable for periods of time due to other priorities or unforeseen circumstances.

#### Technical Knowledge Gap Within Data Governance Approval Teams

Given there was not a defined process, all TREs felt that CO-CONNECT should still apply to the data access teams for access to the data. The paradox though was that the CO-CONNECT team never required access to the data and the whole architecture was designed such that the only teams accessing the data are the teams within the TREs. It was the software, rather than a person, that was accessing the data to enable questions to be answered, which naturally led the data access teams to request more technical details about the software. However, simultaneously, the data security teams within the TREs were independently assessing the software for compliance. The TREs had processes that were more established to assess a typical research project in which a researcher requires access to data, for a particular project. As the application to set up the infrastructure within each data partner was highly technical, it was challenging to find the right level of detail to be both clear and understandable taking into account the knowledgebase of data access teams. In the case of the national TREs, there were different groups or committees assessing the technical and security aspects of the project; however, it did not appear that the data access groups were content to rely on the approval of the technical groups. The data governance committees asked for more technical details, but in some instances, appeared not to have the required expertise to assess the information.

### Different Levels of Technical Capability Across Data Partners

There was a vast difference between the technical capabilities of different data partners. National TREs had different teams of people covering specialist areas, such as data sharing, disclosure control, cyber security, information technology, and data governance, for the CO-CONNECT team to interact with for each technical component. Groups hosting research cohorts of data often only had small teams. The documentation and technical support needed to be tailored to each data partner which took time.

### Production of Linked Extracts of Data From Across Different Databases Held by the Same Organization

Once the data governance had been approved, data partners needed to set up a pseudonymized version of their data for federated querying. For research cohorts of data, this was relatively straightforward, but for national TREs, this was much more complex. Population-level data are held within different databases often managed by different teams within the same organization. The infrastructure was not in place for automated linkage and extraction of data from across these databases. For example, the data we required from Scotland were very similar to the EAVE II dataset [27]. However, we were told that we could not request a copy of that data as it was not a “real” dataset; instead, it was extracts of data from many sources, which would take months to link and clean before being useable. It was unfortunate that we could not reuse the linking and cleaning efforts of others.

### Laboratory Data Feed

Research using granular-level (such as antibody volume and assay type rather than just positive or negative), serology data was key to the COVID-19 response. In the early stages of the pandemic, it was not clear if individuals who had contracted the disease would be immune, and if so, how long that immunity would last. Vendors developed new assays for COVID-19, but data were not easily accessible to calibrate these assays with evidence-driven cutoff ratios. A workstream of the CO-CONNECT project was to collaborate with health boards or trusts to capture granular-level data and to make this data, linked to longitudinal health care records, available for research. Although we discovered that the laboratory systems could be relatively easily configured to capture the additional granular-level data, we found that sending this data onward to national groups collecting population-wide health data was extremely challenging. In England, laboratory data are not routinely collated at a national level and shared with NHS England (the organization that manages other English national datasets), although there is a long-term plan to build such a pipeline. In Scotland, although there is a pipeline set up for health boards to share data with Public Health Scotland (the Scottish national organization that manages relevant Scottish national datasets), they could not accept new laboratory data fields without risking the stability of antiquated systems, and therefore, could not approve boards sending this additional data.

### PPIE on a Highly Technical Project

It was key for CO-CONNECT to include patient and public representatives in the project in a meaningful way. We wanted to ensure their voice was heard in the way that the system was designed and the controls we were applying to protect patients confidentially. However, this was a technical project, and at times, it was challenging to explain complex solutions to a nontechnical audience.

### Lockdown Impacts

CO-CONNECT was initiated during a pandemic when people could not meet in person due to national lockdown restrictions. Outside of a pandemic, such a project would have started with a face-to-face kickoff meeting to get everyone on the same page and to build new relationships. Such a meeting was not possible in person (or even virtually due to the added diary pressures caused by the pandemic). This inability to build relationships and efficiently communicate to everyone at once hampered the timeliness and cohesiveness of the project.

## Solutions and Lessons Learned

### Support for Data Partners

We adapted our approach to support data partners to progress the project while under the added pressures of the pandemic using the following strategies.

#### Regular Short Videoconference Calls

Regular emailing with data partners was generally not as successful for progressing issues as regular “touch base” video calls. The video calls provided a rapid way of addressing any outstanding questions, helped build relationships, and developed or maintained momentum.

#### Standardized Technical Onboarding Pack

Data partners were asking the same questions but in different ways. We initially provided bespoke answers to each data partner based on how they had framed the question. However, over time, we recognized the patterns of the questions and covered the answers in a standardized format within the technical onboarding pack. This was part of the process to scale the onboarding process. We provided the pack as an appendix to all of the different types of governance documentation, and where possible, pointed answers to the questions to the section in the pack rather than bespoke answers [28].

#### Animated Explainer Videos

We worked with a company (Mode13) to develop a series of short, animated videos explaining the design of the federated platform and how results to queries could be securely set externally while protecting data confidentiality [29-32]. Data partners and their data governance committees found that they grasped the solutions much faster than they did reading about them in the abstract. The videos had active input from our PPIE group.

#### Demonstrating the Live System

Demonstrating the system once it was live with the first sets of data also helped data partners earlier on in their journey to understand the benefits of the project and how it could be securely configured.

#### Technical Videos

We developed a series of videos aimed at the data partner technical teams who would be implementing the solution [32]. The technical teams found these videos helped them to grasp the solution quickly and could then follow up with more details within the technical onboarding pack.

#### Workshops and Drop-In Sessions

Bringing data partners together for shared workshops, drop-in sessions, question and answer sessions, and demo sessions helped to develop a shared understanding and confidence in the solution. We also ran a workshop with representatives from the Information Commissioner’s Office and Health Data Research UK (UK National Institute of Health Data Research with expertise in data governance) [33] to address any concerns from the data governance groups. We brought data partner technical teams together for technical workshops with representatives from BC Platforms and the CO-CONNECT technical team.

#### Engaging the Academic Community

Engaging academic data partners (those who had collected data from research cohorts) was achieved by inviting academics to present their research in monthly CO-CONNECT all-partners meetings. This improved understanding of the benefits of the project and the details of the datasets held by the other data partners; developed collaborations; and discussed exemplar research projects that could use the infrastructure.

### Emergence of a 4-Stage Governance Approval Progress for Unconsented Population-Scale Data

Although each national TRE was initially unsure of how to approve the project, a similar 4-stage governance approval process emerged across TRE organizations: a Data Protection Impact Assessment, a data governance request, a Disclosure Control Assessment, and a Security Risk Assessment. Understanding which groups needed to be consulted before the organizations could say “yes” will help similar new infrastructure projects in the future. However, as the groups all required similar information, it could be argued that a more streamlined approach would be for all groups to meet to review the application together based on a single application form containing all the required information. Adoption of the Health Data Research UK standardized data access request form [34] across data partners might have been helpful, however, it was not mature enough to be adopted at the time of the project.

### The Goldilocks Approach to Data Governance Approvals

Different individuals or committees required different levels of detail on application forms. Subsequently, it was difficult to determine the level of detail required at the offset. On many occasions, we were asked to remove details from an application, only to be asked for those same details to be added as part of the reviewer’s response. The lesson learned was that there is a Goldilocks level of detail required. It is unfortunate that Goldilocks’ taste buds were different for each committee within each organization.

We remain unsure of how to address this challenge directly other than perhaps some more guidance throughout the process in the form of a consistent individual within the organization to guide us. This individual could then attend any governance review meetings and answer any questions that may arise to help the approvals process be more efficient and expedient. This process was used within one national TRE for its data access and data governance applications, and it was the only national TRE to complete all tasks and have data accessible via the tool.

### Persistence, Patience, and Understanding

The CO-CONNECT timelines were tight, with the desire to positively impact the COVID-19 response. The delays that we experienced were frustrating, especially for the team directly working with data partners. However, we recognized that all collaborators were under immense pressure from competing demands and still tried their best to support the project. We tried to approach our communications with persistence, patience, and understanding. As with so many things in life, engendering change is hard and takes time, but even more so in the middle of a pandemic.

### Development of a National COVID-19 Serology Laboratory Data Standard

To support the capture of granular-level, serology data, we worked across data partners and 3 different NHS boards or trusts to develop a new serology laboratory data standard. The standard included 12 data attributes providing the required fields for serology research using data from multiple different vendors. Details of the standard are presented in [35].

### PPIE Group

CO-CONNECT had 2 lay coapplicants on the grant proposal. A PPIE group was set up and was heavily involved in all aspects of the project including attendance at workstream meetings. One of the lay coapplicants chaired our monthly all-partners meetings, demonstrating our commitment to PPIE. Another of our PPIE members was a poet and author and wrote several papers covering various aspects of the project including one paper published in a news outlet [36]. PPIE representatives were highly involved in the main project. They attended our workstream meetings and we scheduled time after the core meeting to answer any specific questions they might have and to summarize the conclusions of the meeting to this nontechnical audience. The PPIE group provided helpful feedback and edits to the narrative of the explainer videos and also reviewed the animations—helping us to pitch these videos to nontechnical audiences.

## Recommendations

We recommend 10 key changes to the data landscape so the United Kingdom can respond more quickly to future pandemics and make similar projects more efficient and impactful:

- Single points of failure within organizations that have data and provide access to research could be reduced by training more people in assessing data requests and allowing delegated authority for approvals.
- Having an assigned individual within the organization who is your local “sponsor” proved incredibly useful. Some organizations have it once a project is approved, like the “data buddy” in ALSPAC [37], but having a similar process during the governance approvals could help smooth the process, especially for more complex applications.
- Technical expertise should be embedded within national and regional data governance application panels or committees.
- Data partners should adopt a standard data access request form (such as the one being developed by Health Data Research UK) [34] so that applications in different formats across different data partners are not required. Each application for data to a single data partner should not require multiple forms in different formats for different committees. Targeted information for each group could be provided within appendices rather than requiring the same information to be provided but requested in a slightly different way by each group.
- A light-touch governance process should become standard for the sharing of metadata or profiling data. This data does not contain identifiable information.
- Significant investment should be provided by the government or NHS to enhance data systems across the UK health care system, making them more robust, automated, and scalable. This is not only needed for data for the secondary purposes of research but also to provide timely, high-quality data to respond to clinical and administrative needs. As part of this, data standards adoption should be embedded in the dataset creation process to reduce the requirement for highly specialized, domain expert data engineering which is a limited pool of people who are in high demand in other sectors. If datasets were created to standardized data models, the requirement would be for validation and testing, which can be automated, as opposed to mapping and transformation which requires clinician and engineering input in addition to those steps. The specific standard model is not necessarily an obstacle if its specification is published or available, as mapping from one standard to another is a much less arduous task than mapping from no standard to a standard.
- A national laboratory data feed should be commissioned for NHS boards or trusts to robustly share data with national bodies collecting population-wide data. The infrastructure should be scalable and flexible, supporting additional laboratory tests and data fields as required over time. This should support new laboratory standards as they are developed.
- To modernize the completion of the various governance forms, it would be beneficial if digital portals could be created in place of “paper” forms. These systems could easily provide features such as tracking differences, providing notification of submissions or changes or review, and any comments that have been made by reviewers or collaborators all in one place. They could also provide more guidance and be transparent in showing how the forms are progressing. More than one TRE has a system like this for 1 or 2 forms making the process simpler as everything was more transparent, and it was easier for multiple people to work on the forms simultaneously. It has added benefits of always being up to date with the latest form, and guidance from the host organization, and not falling into the trap of submitting a governance application only to be told that the Word template had changed since you started writing your document. The final benefit is that it provides a single source of truth for your application instead of multiple different versions of the same document circulating between collaborators and reviewers. Health Data Research UK is developing a standardized form and process that can be used across data custodians supporting such needs of the community [38].
- There should be a national process to approve software solutions for use across Health Boards or Trusts and Research Environments. We had to do a security assessment of the software at every location. This challenge could be reduced through a central list of approved or safe software.
- National funding schemes that support innovation in the national infrastructures are vital. The COVID-19 response placed a strain on the existing infrastructures and funding, such as CO-CONNECT, was seen as vital to the response. However, funding for innovation in research infrastructure has previously been hard to secure. The DARE UK program [39] offers a significant opportunity for the technical infrastructure to be given opportunities to innovate and support emerging technologies.

## Long-Term Impacts

Although CO-CONNECT experienced delays, the project was successful in onboarding federated data for cohort discovery. The Cohort Discovery Tool is live within the Gateway and has been queried over 3600 times supporting researchers to carry out feasibility queries across multiple datasets. Data custodians are supported to promote their dataset and researchers can find out about datasets they otherwise would not have known about. At the time of publication, there are 19 research cohorts live within the tool and population-scale datasets from Scotland. NHS England worked with us to test the solution and provide metadata but could not go live with the solutions within the timeframe, due to other competing demands. Data governance has been approved in Wales and the system is ready to go live following final testing.

A new secure architecture has been approved and deployed by >18 organizations hosting both unconsented and consented datasets, which can be enhanced and reused. This new architecture includes new processes for onboarding new datasets, open-source software [21,23-25] to map datasets into the OMOP CDM, open application programming interfaces to enhance and expand the functionality, WhiteRabbit acceptance as software for profiling and producing metadata on sensitive datasets, acceptance of automated disclosure control, federated analytics capability using the same architecture, and datasets that have been converted to OMOP and can be reused by other research projects such as the pan-Europe European Health Data & Evidence Network project [5].

The software and new processes developed by CO-CONNECT will be taken forward by Health Data Research UK, to onboard new datasets covering different diseases; for example, the Alleviate data hub [40] is currently onboarding pain data from across the United Kingdom. The materials developed by CO-CONNECT can be used to onboard new datasets including a range of explainer videos and documentation for data custodians, technical teams, and the general public to understand the solution, benefits, and how we protect patient confidentiality.

The technology has demonstrated the flexibility to be converted to different health care challenges, as it was converted from consented biobanks to be used across large population-level TREs. This flexibility is achieved predominately by the OMOP CDM; therefore, so long as data can be represented in this data model, then the solution can be used for any future pandemic response. CO-CONNECT brought a new federated solution to groups generally unaware of such tools and changed the conversation from *why* we should do cohort discovery or federated analytics to *how* we should. From CO-CONNECT, many other initiatives are now spawning and seeking to embed federation as a core requirement. The work from CO-CONNECT now continues in the Health Data Research UK program under the Federated Analytics Program and has been further accelerated in the DARE UK program, which has accelerated the development of additional federated tooling across different TREs and different domains [41,42]. In England, the NHS Secure Data Environment program is creating regional TREs, and federation across these secure data environments is part of the program.

## Abbreviations

ALSPAC: Avon Longitudinal Study of Parents and Children
CDM: common data model
CO-CONNECT: COVID-19-Curated and Open Analysis and Research Platform
COPI: Control of Patient Information
NIHR: National Institute for Health and Care Research
NHS: National Health Service
OMOP: Observational Medical Outcomes Partnership
PPIE: patient and public involvement and engagement
TDCC: Tissue Directory and Coordination Centre
TRE: Trusted Research Environment
VM: virtual machine
